# Harnessing the effect of iron deprivation to attenuate the growth of opportunistic pathogen *Acinetobacter baumannii*

**DOI:** 10.1128/aac.01689-24

**Published:** 2025-04-09

**Authors:** Sujata Saha, Debasrita RoyChowdhury, Ali Hossain Khan, Sukhendu Mandal, Kunal Sikder, Dipak Manna, Amit Ranjan Maity, Soumyananda Chakraborti, Arnab Basu

**Affiliations:** 1 Department of Biomedical Science and Technology, The School of Biological Sciences, Ramakrishna Mission Vivekananda Educational and Research Institute, Howrah, West Bengal, India; 2 S.N. Bose National Centre for Basic Sciences 30178, Kolkata, West Bengal, India; 3 Department of Microbiology, Ballygunge Science College, Kolkata, West Bengal, India; 4 Institute of Biotechnology, Amity University Calcutta Campus, Kolkata, West Bengal, India; 5 Department of Biological Science, Birla Institute of Technology & Science, Hyderabad Campus 209298, Hyderabad, Telangana, India; Columbia University Irving Medical Center, New York, New York, USA

**Keywords:** *Acinetobacter baumannii*, multidrug resistance, β-Thujaplicin, iron chelator

## Abstract

*Acinetobacter baumannii* is an opportunistic pathogen having high infectivity among immunocompromised patients. The bacteria are resistant to major first-line antibiotics and have become a serious concern in the aspect of nosocomial and community-acquired infections. To overcome this dire situation, the necessity of introducing new approaches is undeniable, which can bypass the need for conventional antibiotic therapy. In this article, we have pinpointed the importance of iron in *A. baumannii.* Iron is an essential micronutrient in all bacteria. Loss of iron acquisition leads to membrane destabilization, and change in the expression of iron-transporting or -metabolizing genes causes death of the bacteria. Iron scavenging was primarily mediated by different chelators, and β-thujaplicin showed the best antibacterial efficacy with respect to time killing assay and CFU analysis. When iron (Fe*^2+^*) was supplemented after initial deficiency, the growth of the bacteria was seen to be restored. Iron deprivation also disintegrates the biofilm matrix, a major cause of bacterial resistance against different types of antibiotics. Moreover, iron scavenging promotes inhibition of biofilm sessile persister cells, the root cause of recalcitrant and chronic infection. As a part of antimicrobial therapy, β-thujaplicin was treated alongside colistin and chloramphenicol at an amount significantly lower than its MIC value. Our results indicated that β-thujaplicin nicely complemented those antibiotics to potentiate their antimicrobial action. In a nutshell, iron chelating agents are potential alternative therapeutics that can be used alongside different antibiotics to circumvent the resistance of different nosocomial pathogens.

## INTRODUCTION

*Acinetobacter baumannii* is an important Gram-negative coccobacillus ubiquitously present in the environment. The World Health Organization has identified *A. baumannii* as one of the most notorious and challenging agents of nosocomial infections worldwide (1). This opportunistic pathogen has been shown to develop resistance to last-line antibiotics, including carbapenems and colistin (2). It is one of the primary causes of local and systemic infections among hospital-admitted patients, particularly those with immunocompromised conditions (3). From molecular and physiological perspectives, the evolution of virulence factors, biofilm formation, and alterations in inherent drug targets are pivotal contributors to antibiotic resistance. Moreover, the formation of persister cells leads to chronic and relapsing infections, further exacerbating the problem. Hence, it is crucial to identify novel therapeutic strategies that can bypass the need for conventional antibiotic therapy (4).

The survival of *A. baumannii* requires several micronutrients (5). Iron is one of the most important trace elements for all bacterial pathogens because of its essential metabolic role (3). It regulates various critical functions, including oxygen transport, respiration, DNA replication and repair, and gene expression (6). In the host, free iron is limited, as it is tightly bound to different biomolecules, such as heme (7). Similarly, under physiological conditions, the availability of free iron remains extremely low due to the rapid oxidation of Fe²^+^ to Fe³^+^ and the subsequent formation of insoluble metal hydroxides (8). Since the concentration of Fe³^+^ under these conditions is very low (10⁻⁹ M), bacteria face significant challenges in acquiring iron (9). Given that iron acquisition is crucial for the virulence and survival of *A. baumannii* within the host, it is essential for the bacterium to obtain iron from the iron-limited host environment (10). To adapt to different host environments, *A. baumannii* modifies its metabolic processes by employing specialized mechanisms for nutrient acquisition (5). Alternatively, the host maintains a very low extracellular iron concentration by sequestering it inside cells and producing metal-binding proteins, such as transferrin, lactoferrin, and the oxygen-carrying protein hemoglobin (11). These defensive strategies significantly reduce the supply of free iron, which is essential for pathogen survival. This host defense mechanism is known as nutritional immunity (12).

A major iron uptake pathway involves the direct binding of Fe²^+^ or heme to receptors or other transporter proteins on the cell surface. Additionally, a more energy-intensive iron acquisition mechanism involves the production and secretion of high-affinity iron-chelating molecules, known as siderophores, which are low-molecular weight compounds that compete with host cells by scavenging extracellular iron (13). Acinetobactin, Baumannoferrin, and Fimsbactins are three distinct classes of siderophores identified in *A. baumannii* (10). Acinetobactin exhibits a strong affinity for iron and employs the BauA receptor on the outer membrane, which is TonB-dependent (14). This receptor utilizes a proton-motive force and is supported by an inner-membrane ATP-binding cassette (ABC) transporter complex comprising BauB, BauC, BauD, and BauE. Together, these components facilitate efficient iron uptake into the bacterial cell. On the contrary, the siderophores Baumannoferrin and Fimsbactins exhibit a high affinity for iron and employ the TonB-dependent outer-membrane receptors, BfnH and FbsN, respectively. These receptors similarly harness the proton-motive force to mediate iron transport into the cell (15).

Recent research into microbial iron acquisition using iron chelators has identified promising opportunities for developing advanced antimicrobial strategies (16). In this article, we aimed to examine the mechanism of action of several iron chelators on the growth physiology of *A. baumannii*. We used different synthetic iron-chelating agents, such as 2,2′-dipyridyl (DIP), hinokitiol (β-thujaplicin), and deferoxamine (DFO) (17, 18). Regarding antimicrobial efficacy, the iron chelator β-thujaplicin showed greater efficiency in killing the planktonic form of the bacteria and inhibiting biofilm formation, which is pivotal for nosocomial infection. Additionally, this synthetic iron chelator was effective against persister cells, significantly reducing their number under iron-deprived conditions. While investigating the mechanism, it was observed that iron depletion leads to an increase in cell membrane permeability, allowing the inflow of environmental materials, which damage the cell. Iron chelation also alters the expression of iron**-**regulating and stress-responsive genes in *A. baumannii*, which has important consequences for iron homeostasis. In summary, our study presents an alternative therapy to overcome multidrug-resistant nosocomial pathogens, with a plausible mechanism of action for β-thujaplicin.

## RESULTS

### Antibacterial effect of different chelators on *A. baumannii*

**Fig 1:**
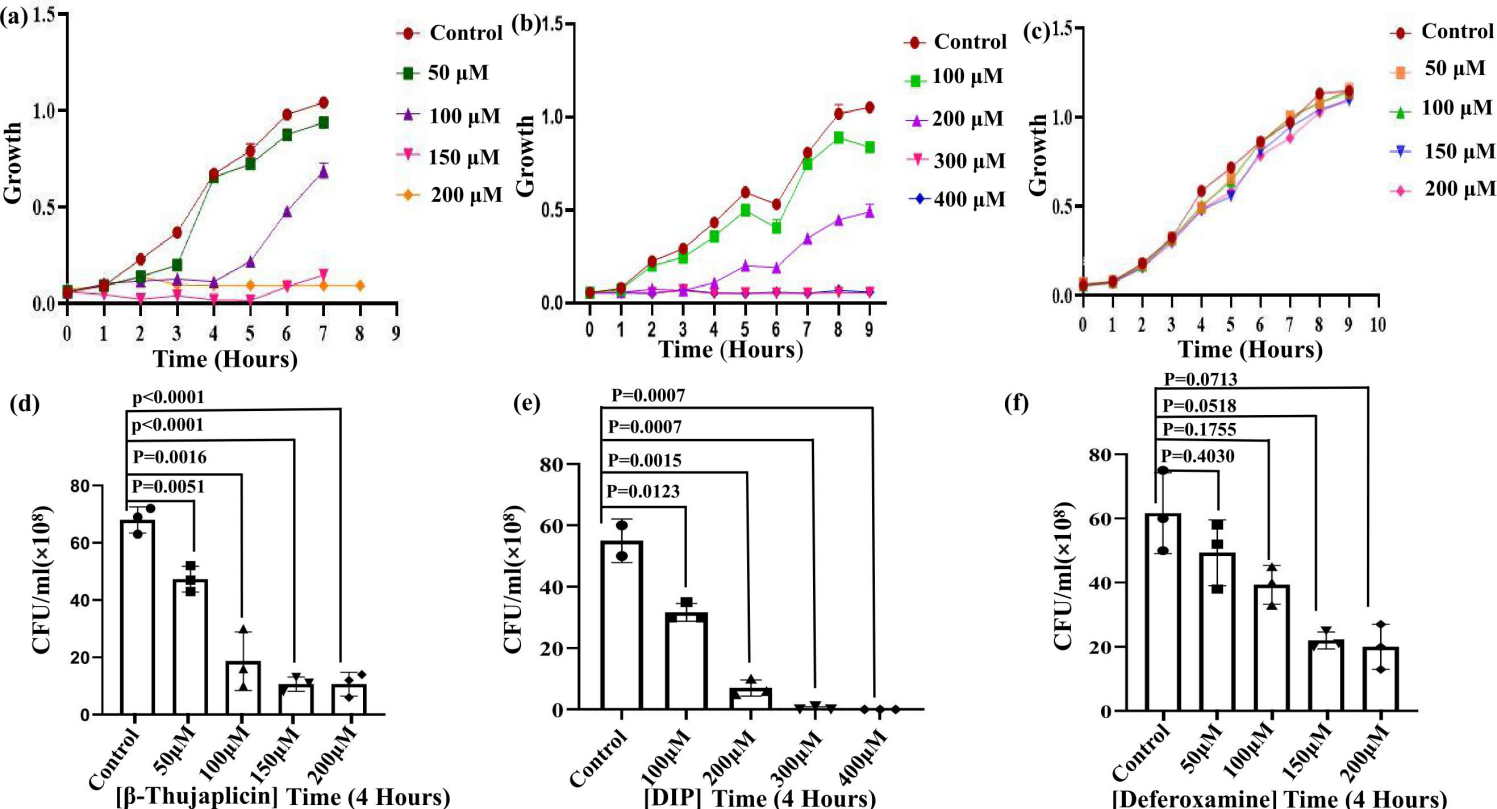
Antibacterial effect of different chelators on *Acinetobacter baumannii*. (a) Growth curve analysis revealed that bacterial growth was inhibited up to 9 h upon treatment with 50–200 µM β-thujaplicin. (b) Bacterial population analysis in the presence of 100–400 µM DIP showed significant growth inhibition at 300–400 µM. (c) Bacterial growth was assessed following sequential treatment with 50–200 µM deferoxamine (DFO). (d) The bacterial growth curve results were validated using CFU analysis, which demonstrated a statistically significant reduction at the 4 h time point, confirming the growth curve findings. Additionally, bacterial colony numbers were markedly reduced after sequential β-thujaplicin treatment. (e) In the presence of DIP, bacterial cell viability decreased compared to the control. (f) CFU enumeration was performed in the presence of DFO. The experiment was repeated three times, and data were analyzed using GraphPad Prism represented as the standard error of the mean (± SEM). Statistical significance was determined using Student’s *t*-test. Different symbols have been used to mark different samples in the graph used in survival analysis.

To ascertain the ideal growth conditions for the bacteria, the growth of *A. baumannii* MTCC DS002 was observed under different concentrations of iron chelators. Initially, we checked the effect of the quenchers on the growth of *A. baumannii*, followed by the enumeration of colony-forming unit (CFU) to assay bacterial survival. In each case, we measured the growth profile for up to 9 h with sub-lethal to lethal concentrations of quenchers. To have a prior idea about the concentration**,** we performed a minimal inhibitory concentration (MIC) assay (19). Our growth curve analysis showed that bacterial growth was most efficiently inhibited in the presence of β-thujaplicin (Fig. 1a). In the presence of 100 µM of β-thujaplicin, which is a lower concentration than that of other chelators, bacterial growth was drastically reduced. In the case of another chelator, such as DIP, it had an intermediate effect with partial loss of viability at a concentration of 200 µM (Fig. 1b). Conversely, DFO had no obvious effect on the growth of the bacteria (Fig. 1c). The expected number of viable bacterial cells was drastically reduced following treatment with a lethal dose of β-thujaplicin, as demonstrated by our CFU analysis at the 4 h time point (Fig. 1d). Our bacterial viability analysis showed that 100 µM of β-thujaplicin exhibited maximum inhibition compared to other quenchers (Fig. 1e and f). Thus, iron is indispensable for bacteria, and the reduction of iron in the growth medium significantly impacts bacterial growth and physiology. In summary, a careful regulation of intracellular iron concentrations is pivotal for bacterial survival. Trypan blue staining was performed to assess the killing efficiency of iron chelators in real time (Fig. S1a). Trypan blue can penetrate dead cells through pores developed in the membrane. Iron-deprived bacterial cells appeared violet, as they took up the stain thoroughly, while most of the bacteria in the untreated sample did not take any stain. We further validated our experiment by resazurin assay, which uses a metabolic dye that changes color from purple to pink upon oxidation in the presence of an enzyme found in live bacteria. The sample treated with iron chelators showed no visible color change, remaining purple. In case of the control, the dye completely changed to pink (Fig. S1b).

### Reduction of iron increases cellular permeability and reduces reactive oxygen species production

**Fig 2:**
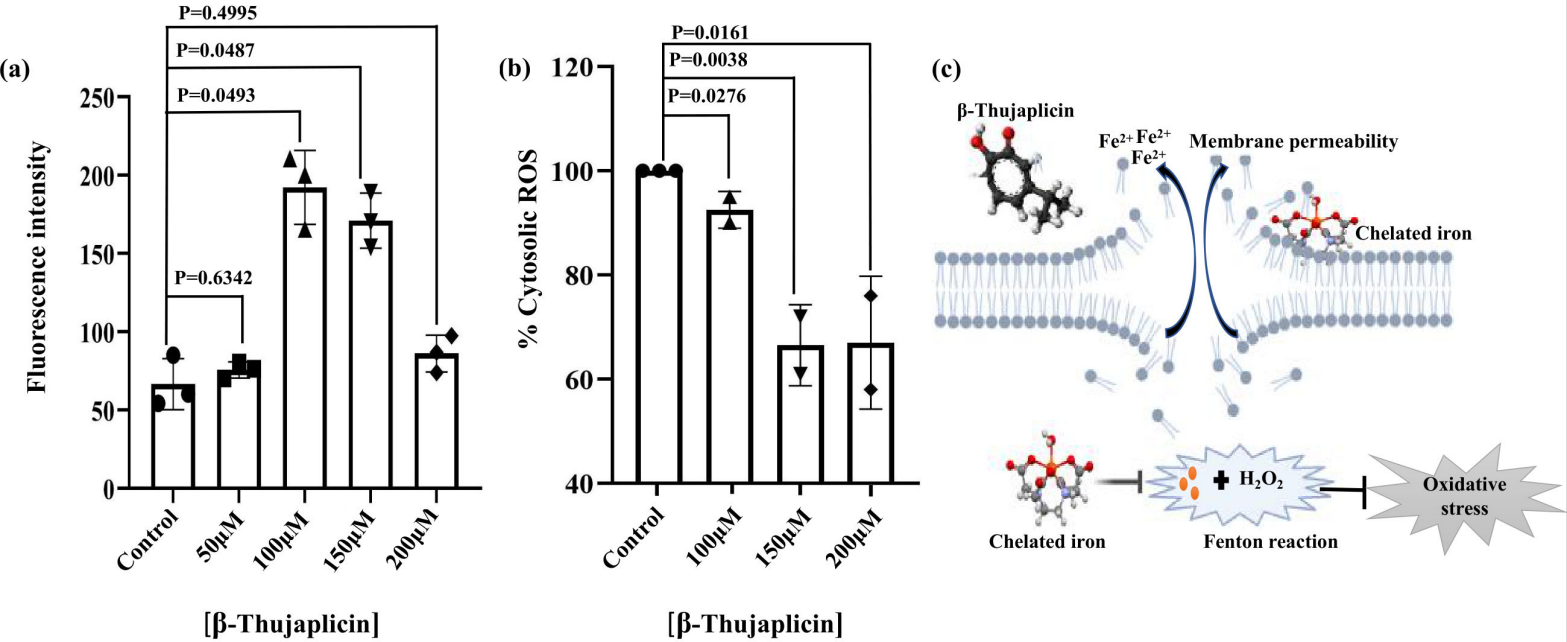
Reduction of iron increases cellular permeability and reduces ROS production. (a) The cell membrane permeabilization assay revealed a significant increase in membrane permeability in the presence of varying concentrations of β-thujaplicin. (b) β-Thujaplicin treatment led to a dose-dependent decrease in cytosolic ROS production, with higher concentrations of the iron chelator further reducing the ROS levels. All experiments were performed in triplicate, and data were analyzed using GraphPad Prism represented as the standard error of the mean (± SEM). Statistical significance was determined using Student’s *t*-test, with *P* < 0.05 considered significant. (c) A proposed model illustrates the effect of β-thujaplicin on *Acinetobacter baumannii*, showing that iron chelation increases cell membrane permeability by removing divalent cations from the outer membrane. Additionally, iron chelation inhibits the Fenton reaction, resulting in reduced cytosolic ROS production compared to untreated cells. For indicating different samples, the same trend was followed.

The chelator β-thujaplicin showed inhibition of bacterial growth in the planktonic state when applied at a sub-lethal concentration. Since the effect is primarily mediated by the sequestration of iron from the environment surrounding bacteria, we were interested to know how it affected bacterial cells. As the outer membrane is the outermost barrier in Gram-negative bacteria, we first checked the effect of iron depletion on the integrity of the structure by measuring cellular permeability. As our results suggested, we observed a drastic cellular alteration characterized by increased cellular permeability in response to increasing concentration of β-thujaplicin. The effect was manifested by the increased fluorescence intensity of 1-N-phenylnaphthylamine (NPN) inside the cellular compartment as it entered the cell through the pores in the membrane. However, an increase in cell membrane permeability was evident only up to 100 µM of β-thujaplicin (Fig. 2a). Using higher concentrations of the chelator resulted in a large pore formation, leading to rapid cell lysis. It can be assumed that β-thujaplicin (hinokitiol) may selectively chelate out Fe²^+^ from the surface of the phospholipid bilayer, thus interfering with the negative charge stabilization of phospholipid molecules in the outer membrane. The increase in permeability may result from its incorporation into the phospholipid structure or the reduction of a particular component of the outer membrane, as seen in a previous analysis (17). Consequently, we observed the loss of NPN fluorescence, which indicated cell lysis. Alternatively, the production of reactive oxygen species (ROS) has been investigated. Iron (Fe²^+^) is an essential component for controlling the Fenton reaction, which ultimately leads to hydroxyl radical generation, a major source of ROS in the cytosol. Iron sequestration results in loss of iron import, thereby reducing the effective amount of iron available for the Fenton reaction. As a consequence, we observed a reduction in ROS levels in the bacterial cell at increasing concentrations of β-thujaplicin (150 µM and above) (Fig. 2b). Since the 2′−7′-dichlorodihydrofluorescein diacetate (DCFDA) treatment was performed for 2 h on mid-log-phase grown cells, the reduction in ROS suggests a decreased availability of cellular iron in *A. baumannii*. Thus, it can be concluded that the loss of cellular viability is not due to intracellular oxidative stress but rather an outcome of membrane structural destabilization caused by inadequate Fe²^+^ on the membrane surface of the bacteria. The inherent negative charge of the phospholipid in the outer membrane is counterbalanced by divalent cations, which are lost due to intracellular iron metabolism disruption. As a result, the outer membrane becomes prone to distortion, leading to an influx of external materials into the cytosol, causing osmotic lysis.

### Effect of iron starvation on biofilm and persister cell

**Fig 3:**
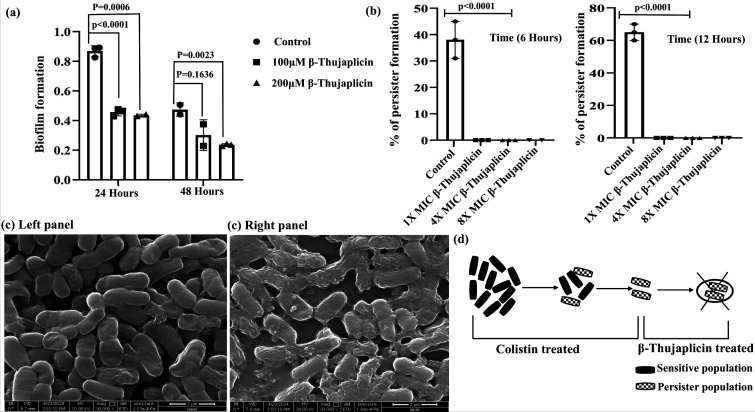
Effect of iron starvation on biofilm formation and persister cells. (a) Biofilm quantification at 24 and 48 h in the presence of β-thujaplicin demonstrated a statistically significant reduction in biofilm formation due to iron chelation. Experiments were performed in triplicate, and statistical significance was determined using Student’s *t*-test (*P* < 0.05). The variation in concentration has been indicated with different symbols shown in the graph. (b) The impact of β-thujaplicin on persister cell formation was assessed at 6 and 12 h post-treatment with 1×, 4×, and 8× MIC of the compound. A negligible percentage of persister cells was observed compared to colistin treatment. (c) Scanning electron microscopy (SEM) analysis showed the characteristic coccobacillus morphology of *A. baumannii* in the untreated condition (left panel). However, treatment with 200 µM β-thujaplicin led to extensive cellular aggregation and leakage of intracellular material under iron-starved conditions (right panel). All SEM images were captured within a 2 µm range. (d) A proposed model illustrates the inhibition of persister cell formation in the presence of β-thujaplicin and colistin.

Biofilm is an essential component for bacterial survival amid environmental assaults and antibiotic stress. Nosocomial pathogens, such as *A. baumannii*, use this component as a medium of infection in clinical settings. In this study, we evaluated the effect of iron on biofilm formation. Previous research has shown that divalent cations, including Fe²^+^, are essential for maintaining the integrity of the biofilm matrix. When iron sequestration occurred in the presence of increasing concentrations of β-thujaplicin (100 and 200 µM), a gradual loss of biofilm was observed at 24 and 48 h, respectively (Fig. 3a). We focused our analysis on the generation of persister cells, which exist within the biofilm matrix. Persister cells hold paramount importance because they contribute to chronic and recalcitrant infections, even after antibiotic therapy is completed, posing a significant challenge in the hospital sector. Since they exist in the innermost core of the biofilm, they exhibit greater resistance to antibiotics than other cells. Due to their limited access to oxygen and nutrients, persister cells have very low metabolic activity and, therefore, can only be destroyed when the biofilm matrix is interrupted. Here, we examined the effect of the chelator β-thujaplicin on the formation and destruction of persister cells. Disrupting the matrix affected the generation of persister cells. A complete loss of persistence was observed in the presence of a lethal concentration of iron chelators at 6 and 12 h (Fig. 3b). The effect of iron sequestration was severe, and complete inhibition was observed in the presence of 200 µM β-thujaplicin, which is lower than its MIC value for *A. baumannii* (Fig. S3a). We also investigated the effect of β-thujaplicin on *A. baumannii* in real time using a scanning electron microscope (SEM). Exposure to a threshold lethal dose of β-thujaplicin (200 µM) resulted in the loss of cellular structure and large aggregates of cellular mass, which were visualized under the electron microscope (Fig. 3c). The effect was initiated by membrane perforation, which led to the loss of cellular materials, causing such aggregation during iron-starved conditions.

### Rescue of bacterial survival and cytosolic ROS generation after supplementation of iron

**Fig 4:**
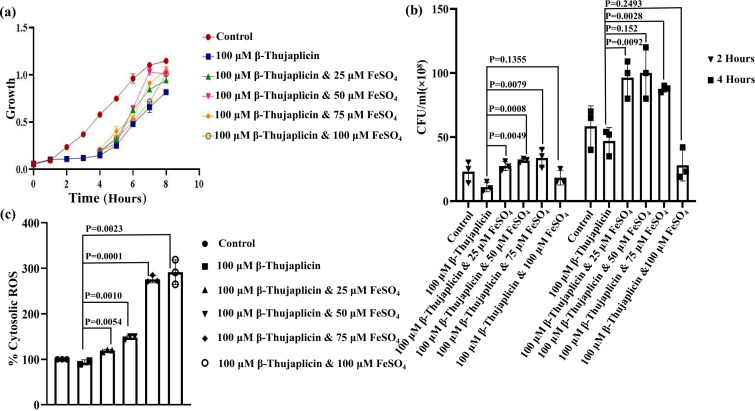
Restoration of bacterial growth and cytosolic ROS generation following iron supplementation. (a) Under iron-deprived environment; bacterial growth was monitored following the sequential addition of 25, 50, 75, and 100 µM FeSO₄ over 9 h of time. Significant bacterial growth recovery was observed with 50 µM FeSO₄ supplementation, while the 100 µM β-thujaplicin-treated condition served as the control. (b) Bacterial survival was further evaluated by CFU analysis at 2 and 4 h post-iron supplementation, with maximal bacterial recovery observed at 50-75 µM FeSO₄. Samples from different time points have been denoted with different symbols. (c) Cytosolic ROS levels were assessed following 100 µM β-thujaplicin treatment and subsequent supplementation with 25, 50, 75, and 100 µM FeSO₄ in iron-depleted media. Oxidative stress levels increased with higher FeSO₄ concentrations. All experiments were performed in triplicate, and data were analyzed using GraphPad Prism (± standard error of the mean). Statistical significance was determined using Student’s *t*-test (*P* < 0.05). Different concentrations have been marked with different symbols.

From our previous analysis, it was found that β-thujaplicin is a potent iron chelator, and it exhibits antibacterial activity against the opportunistic pathogen *A. baumannii* by chelating out iron. After administration of 100 µM β-thujaplicin, bacterial growth was retarded, as suggested by the growth curve and CFU analysis. Here, we examined the rescue of bacterial growth by supplementing iron (Fe²^+^) after iron chelation. After 3 h of 100 µM β-thujaplicin treatment, bacterial cells were supplemented with different concentrations of FeSO₄ (i.e., 25, 50, 75, and 100 µM). The results indicated a revival of bacterial growth after treatment with various concentrations of FeSO₄ (Fig. 4a). Growth enhancement was observed after 1 h of supplementation, and it continued until the end of our experiment. Partial growth restoration was evident from the growth curve analysis. When cell viability was validated by the CFU study, an interesting observation was made. The number of CFUs increased proportionally up to 75 µM, but a slight loss of viability was seen in the case of the 100 µM FeSO₄-supplemented sample at both 2 and 4 h time points (Fig. 4b). This loss of survival may be due to iron toxicity inside the cellular compartment, which induces oxidative stress. An excessive amount of Fe²^+^ may cause the overproduction of OH· by triggering the Fenton reaction. To justify our assumption, we checked the production of intracellular ROS using DCFDA. ROS production was observed to increase with the concentration of supplemented FeSO₄ up to 100 µM (Fig. 4c). As a major micronutrient, supplementation of Fe²^+^ boosted the growth of *A. baumannii* up to 50 µM. However, an excess of Fe²^+^ led to overproduction of ROS, resulting in lipid peroxidation, protein aggregation, or DNA damage, ultimately leading to cell lysis.

### Expression of iron-regulating genes in response to iron starvation

**Fig 5:**
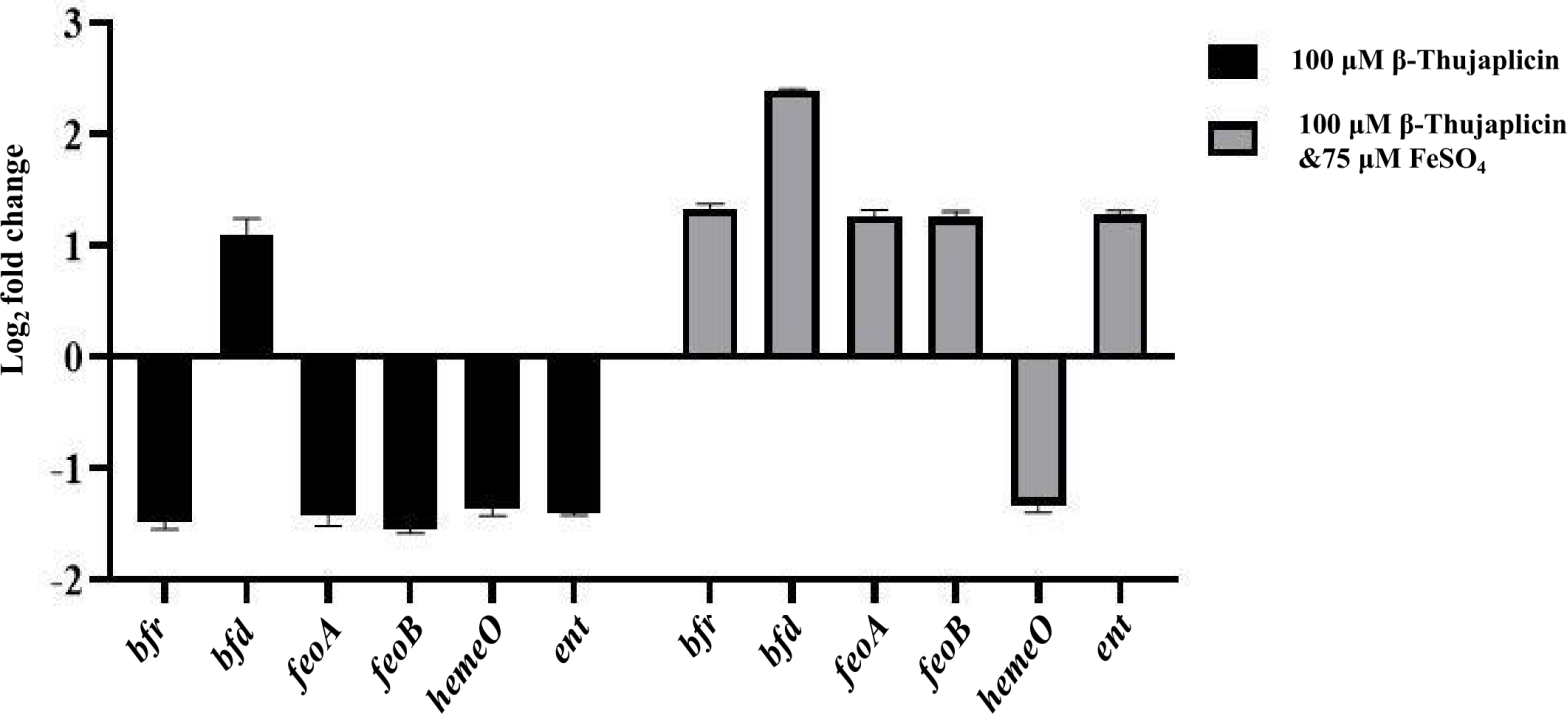
Gene expression analysis. Expression of iron-regulating genes in *A. baumannii* performed under iron-limiting conditions (100 µM β-thujaplicin) and after iron supplementation (75 µM FeSO₄). Under iron-chelated conditions, genes encoding iron storage proteins and iron transporters were downregulated, whereas the *bfd* gene was upregulated. Iron supplementation reversed this effect, leading to the upregulation of iron transport-associated genes.

Iron homeostasis in *A. baumannii* is controlled by different transporters and cytosolic proteins. Here, we illustrate the expression of different proteins of the nosocomial pathogen involved in iron metabolism under normal and iron (Fe²^+^)-deprived conditions (Fig. 5). The Feo system is a crucial component for iron uptake in Gram-negative bacteria (20). FeoB is the major protein for the uptake of iron into the cell and plays an important role in the pathogenesis of *Clostridium perfringens* (21). Bacterioferritin is an essential cytosolic protein that helps in iron storage (6). Bacterioferritin-associated ferredoxin (Bfd) is another protein that works in tandem with bacterioferritin, providing a reducing equivalent to Fe³^+^ stored in bacterioferritin, thus mobilizing iron in the cytosol for bacterial utilization (22). Besides that, *A. baumannii* produces different siderophores for the acquisition of iron from the environment (15). The expression of a repertoire of siderophores, transporters, and proteins associated with iron solubilization was analyzed using RT-PCR. In the presence of a sub-lethal dosage of β-thujaplicin (100 µM), all the regulatory gene expression was reduced to a certain extent, except Bfd. Bfd plays a crucial role in solubilizing Fe³^+^ to Fe²^+^ in the cytosol from bacterioferritin (Bfr), which serves as an iron storage protein. Since Bfr expression remains unchanged in the absence of iron from the external environment, Bfd expression was observed to increase. All other iron regulatory genes were suppressed as the available iron was sequestered in the presence of β-thujaplicin. Next, when we supplemented iron in the form of FeSO₄ at a non-toxic concentration, we observed the overexpression of all iron-regulatory genes used in the earlier set, except heme oxygenase (hemeO). This particular protein is expressed during host infection as it helps in extracting iron from the heme moiety of hemoglobin. Due to the inactivity of this protein outside the host system, downregulation of heme oxygenase was noted. Moreover, heme oxygenase participates in Fe³^+^ transport, which was not required in this study. Understandably, the expression of Fe²^+^-carrying transporters (such as feoA-feoB) was elevated (23). Incidentally, Bfr catalyzes the oxidation of cellular Fe²^+^ to the ferric state and sequesters it in the central cavity of the nanocage; thus, its expression was upregulated. Similarly, bfd expression was also increased. All these results demonstrate that *A. baumannii* modifies its gene expression under iron-limiting conditions to maintain its survival. Severe iron deficiency impairs homeostasis and ultimately causes bacterial death.

### β-Thujaplicin showed synergy with colistin in inhibiting the survival of clinically resistant strains of *Pseudomonas aeruginosa*

**Fig 6:**
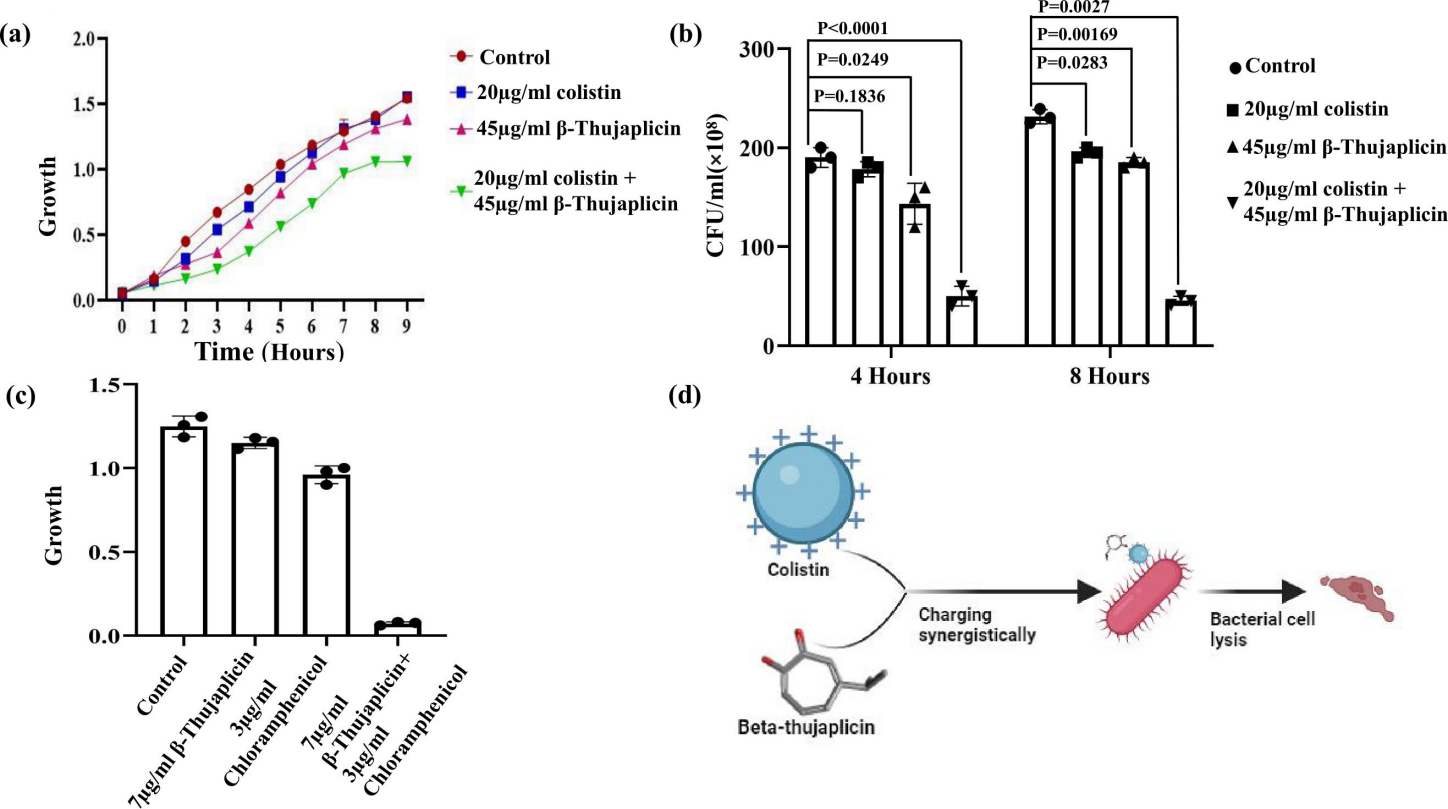
Synergistic effect of β-thujaplicin and other antibiotics in inhibiting *P. aeruginosa* and *A. baumannii* growth. (a) The synergistic interaction between β-thujaplicin and colistin in *P. aeruginosa* was assessed through growth curve analysis over 9 h. A significant reduction in bacterial growth was observed with 20 µg/mL colistin and 45 µg/mL β-thujaplicin, indicating synergy. (b) Bacterial growth inhibition was further validated by CFU enumeration. (c) The synergistic effect of β-thujaplicin with chloramphenicol was confirmed by MIC analysis in *A. baumannii*, demonstrating that 7 µg/mL β-thujaplicin and 3 µg/mL chloramphenicol effectively eradicated bacterial cells. (d) A proposed model explains the synergy between iron chelators and antibiotics, illustrating how membrane destabilization by β-thujaplicin enhances antibiotic penetration, thereby potentiating the bactericidal effect.

*Pseudomonas aeruginosa* is another important member of the ESKAPE group and poses a significant threat in clinical settings. The major problem in treating this pathogen arises due to its resistance against several classes of antibiotics, including the last-resort antibiotic colistin. In our laboratory, we have an extremely colistin-resistant clinical strain of *P. aeruginosa*, which also exhibits resistance against other classes of antibiotics. Since our clinically isolated *A. baumannii* did not show as much resistance to colistin, we switched to analysis with *P. aeruginosa* in this aspect. The MIC of *P. aeruginosa* in the presence of colistin is 5,000 µg/mL, which is quite a high concentration. *A. baumannii* showed far less resistance to the same antibiotic. The scenario was almost the same in the case of chloramphenicol, which exerts an inhibitory effect on bacteria by suppressing the protein synthesis machinery. While conducting a combined application of different antibiotics and the chelator β-thujaplicin against *P. aeruginosa*, we administered at least five times less than or even lower concentrations of the drugs compared to their separate application. The MIC value of β-thujaplicin against *P. aeruginosa* was determined to be 230 µg/mL in our observation. In both growth curve analysis and CFU determination studies, we found a significant reduction in bacterial growth with colistin and β-thujaplicin (Fig. 6a) . The presence of a small amount of β-thujaplicin (45 µg/mL) amplified the effect of colistin by several orders of magnitude (almost 250-fold) in preventing bacterial growth. A distinct difference in the growth curve was observed, while more than an 80% reduction in viable cells was evident in CFU analysis (Fig. 6b). A similar phenomenon was observed in the case of the β-thujaplicin–chloramphenicol combination. A concentration of 7 µg/mL of β-thujaplicin enhanced the activity of chloramphenicol by 10-fold, as evidenced by our MIC analysis (Fig. 6c). The use of lower concentrations of antimicrobials is beneficial for therapeutic applications, as it minimizes toxicity inside the host. In fact, we did not observe any cytotoxic effect of β-thujaplicin in mammalian cell line C2C12, even at 8x higher concentrations than its MIC value (Fig. S2). Hence, it can be concluded that the iron chelator β-thujaplicin is a promising option for antimicrobial therapy and can be used in combination with other antibiotics to enhance their efficacy. The use of chelators alongside antibiotics to restrict the growth of bacterial pathogens is not uncommon and has been reported in previous studies (24). The observed synergistic inhibition is likely driven by outer membrane perforation due to Fe²^+^ depletion, which enhances the penetration of antibiotics into the bacterial membrane, leading to a greater bactericidal effect when applied in combination.

## DISCUSSION

*A. baumannii* is one of the most prominent threats in modern healthcare systems. With the application of an increasing number of immunocompromised hosts and invasive techniques, the pathogen has become endemic in clinical settings. The pathogen has the remarkable ability to tolerate harsh environments and quickly produce resistance factors that help it develop resistance against several antibiotics. All four classes of β-lactam antibiotics have been seen to be resistant to different strains of *A. baumannii* due to the presence of several classes of β-lactamase (25). Numerous acetyltransferases, phosphotransferases, and adenyltransferases modify aminoglycoside antibiotics, thereby rendering resistance against those antibiotics (26). Multidrug-resistant efflux pumps have been shown to have roles in bacterial pathogenicity, showing resistance against tigecycline or imipenem (27, 28). Alternating target sites or loss of cellular permeability are the other consistent resistant mechanisms seen in opportunistic pathogens (29, 30). Most importantly, those resistance factors are disseminated from one strain to another by horizontal gene transfer (31). Unfortunately, the last-resort antibiotic colistin is also shown to be resistant to certain classes of *A. baumannii*, which used to be the wonder drug combating infection of the bacterial pathogen (2). As per a recent investigation, the pipeline of novel antibacterials is still empty and will remain so for a considerable period (32). In this present context, a more holistic preventive measure is required to control the infection of multidrug-resistant *A. baumannii*.

Owing to the increasing antibiotic resistance, a promising approach is to harness the efficiency of iron utilization in this opportunistic pathogen. Bacterial pathogens produce siderophores when they sense the limitation of free iron available in the medium (33). Since iron is an indispensable component regulating multifaceted biological functions (i.e., DNA biosynthesis, oxygen transport, cell respiration, and gene regulation), deficiency of this essential micronutrient affects bacterial virulence. Nutritional immunity is such a phenomenon seen during a pathogen’s infection in the mammalian host. So, the importance of siderophores is ever-growing, and perturbation of their action leads to severe problems in iron acquisition and concomitant impairment of bacterial metabolism. Iron-chelating agents indirectly circumvent the action of bacterial siderophores, as the effective amount of available free iron is taken away from the usage of the pathogen. Quorum sensing (QS) helps in generating siderophores under the limitation of iron (34). By applying QS, bacteria can delay their transformation to a sessile state from the planktonic state. Under severe low iron conditions, biofilm formation is triggered, where they can share iron and other nutrients, slowing down their metabolism substantially. The application of iron chelators not only reduces the effective amount of iron to planktonic cells but also interferes with the formation of biofilm, as iron (Fe²^+^) and other divalent cations are instrumental for the biofilm matrix. This, in turn, creates an extremely adverse situation for the bacteria, which is almost impossible to overcome.

Another degree of resistance is shown by the bacterial pathogens through the formation of persister cells. Bacterial persisters can tolerate high levels of antibiotics and thus cause recalcitrant infections, worsening the problem of antibiotic resistance. Persister cells can be induced by the toxin–antitoxin system in the planktonic stage, or they can also be regulated during biofilm-sessile conditions under antibiotic stress (35). An earlier study showed that iron might play a role in the inhibition of persister cells. Successive application of meropenem and an iron chelator could effectively destroy persister cells in *Escherichia coli*, whereas the combined therapy alone failed to produce such an effect (36). Our analysis features an almost similar readout in this context. The consecutive application of colistin and β-thujaplicin inhibited the formation of *A. baumannii* persister cells. Perhaps, the persister cells are exposed to the antimicrobials directly before maturation, as the biofilm matrix is disrupted due to iron depletion in the medium. However, the scenario can be different in the case of persister cells derived from a planktonic state, which show considerable resistance against antibiotics or other antimicrobials (24). Perhaps, the state of exposure to the antimicrobial is important for the inhibition or generation of persister cells located at different states. The mechanism of iron chelator β-thujaplicin-mediated inhibition of persister cells will be a fascinating area of research that can be explored in the future.

Antibiotic action in iron-limited conditions is a popular combination to treat nosocomial pathogens, as manifested in various literature. Thiostrepton, a Gram-positive thiopeptide antibiotic, produced synergy with the iron chelator deferasirox to prevent *P. aeruginosa* and *A. baumannii* isolated from clinical samples (37). Hydroxypyridinone hexadentate-based dendrimeric chelators potentiated the bactericidal efficacy of norfloxacin in Gram-positive *Staphylococcus aureus* (38). Our result in this context shows that β-thujaplicin exhibits definite synergism with colistin and chloramphenicol in restricting *P. aeruginosa* growth, apart from its bactericidal effect on *A. baumannii*. The loss of iron (Fe²^+^) destabilizes the phospholipid-made outer membrane, which aids in the penetration of the antibiotic through the membrane to reach inside the bacterial cell to register its action. This incident avoids the permeability issue and promotes antibiotic action directly. The combination of an iron chelator and antibiotics, thus, bypasses various specific resistance mechanisms of *A. baumannii* and can be used as a potential therapeutic strategy to alleviate antimicrobial resistance.

## MATERIALS AND METHODS

### Materials

A bacterial strain of *A. baumannii* (DS002) was obtained from MTCC. Luria broth, crystal violet, NPN, thiobarbituric acid (TBA), glutaraldehyde, colistin, chloramphenicol, dimethyl sulfoxide (DMSO) were purchased from SRL. β-Thujaplicin, FeSO₄, DCFDA, and TBA were received from Sigma Aldrich. RNA isolation kit (GR1001), cDNA synthesis kit (G4641A), Sybr Green, and ROX were purchased from GCC Biotech.

### Methods

#### Determination of minimum inhibitory concentration

The MIC is significant, as it provides crucial insights into the optimal dosage of quenching agents for their effectiveness. According to the Clinical and Laboratory Standards Institute, MIC was determined by the broth microdilution method (39). The overnight culture of *A. baumannii* was taken and adjusted accordingly so that each tube contained 10⁵ cells. The whole experiment was done in a 96-well plate with a total volume of 200 µL. A gradually increasing concentration of β-thujaplicin (80–260 µM) was added to the bacterial cultures. Similarly, to determine the MIC of DIP (100–400 µM), chloramphenicol (1–30 µg/mL), and colistin (1,000–5,000 µg/mL) were added to bacterial culture, and the mixture was incubated overnight in a shaker incubator at 37°C. Optical density (OD) was measured at 600 nm. The experiment was done in triplicate, and the results were analyzed using GraphPad Prism software.

#### Analysis of bacterial growth and CFU count

Overnight-grown saturated culture of *A. baumannii* was inoculated in Luria–Bertani (LB) medium at a 1:100 ratio and grown in the presence of different concentrations of iron-chelating agents ( β-thujaplicin, DIP and deferoxamine). The culture was placed in a shaker incubator at 37°C at 200 rpm. The optical density of the culture was measured at 600 nm every hour to monitor the growth profile of *A. baumannii*. The cultures were collected at a specific time point (4 h), serially diluted to 10⁶ cells using 1× phosphate-buffered saline (PBS), and CFUs were enumerated based on absorbance differences. The difference in CFU reflects the relative abundance of live cells in the culture, which corresponds to the growth of the organism at that time. The experiment was done in triplicate, and the results were plotted using GraphPad Prism software. Statistical analysis was performed using a paired *t*-test.

#### Biofilm quantification in the presence of an iron chelator

Overnight culture of *A. baumannii* was prepared as described earlier and incubated in the presence of 100 and 200 µM of β-thujaplicin at 37°Cfor 24 and 48 h, respectively, in an incubator shaker at 200 rpm. Biofilm quantification was performed in a 96-well plate. Planktonic cells were removed, and the plates were air-dried. Then, 100 µL of methanol was added to each well for fixation. Methanol was then removed, and the plates were washed with 1× PBS and left to air dry. To quantify the total biofilm, 100 µL of 0.1% crystal violet was added and incubated for 15 min at room temperature. Excess, unbound dye was washed away with water and air-dried again. To measure biofilm-bound crystal violet, 100 µL of 33% acetic acid was added to dissolve the biofilm mass. Finally, absorbance was measured at 575 nm (40). The assay was analyzed using GraphPad Prism with an unpaired *t*-test.

#### Analysis of bacterial membrane permeabilization

The overnight-grown culture of *A. baumannii* was diluted up to 10⁶ CFU/mL and treated with subsequent concentrations of β-thujaplicin (i.e., 50, 100, 150, and 200 µM). Then, it was kept in a shaker incubator at 37°C for 18 h. After the incubation, cells were harvested by centrifuging at 3,000 rpm for 10 min and washed with 50 mM PBS (pH 7.4). The mixture was resuspended in the same buffer and adjusted to OD₆₀₀ = 1.0. After that, 20 µM of N-phenyl-1-naphthylamine (NPN; Sigma-Aldrich, Steinheim, Germany) was added to 100 µL of the cell suspension, and fluorescence intensity was measured immediately with the fluorimeter (F-7000) at excitation and emission wavelengths of 350 and 420 nm, respectively (41). The experiment was performed twice, and the statistical significance was determined using an unpaired *t*-test comparing the control and the treated sample.

#### Measurement of the generation of reactive oxygen species

The presence of cytosolic reactive oxygen species was illustrated using DCFDA (2′−7′-dichlorodihydrofluorescein diacetate) dye. Bacterial cultures were prepared as mentioned earlier and grown up to the late-log phase, where 800 µL of the cell suspension was treated with different concentrations of β-thujaplicin (50 and 100 µM). The mixture was incubated for 1 h at 37°C. The cytotoxicity of *A. baumannii* was determined by adding 5 µM DCFDA dye as the final concentration. The mixture was incubated at 37°C for 30 min in the dark, and fluorescence intensity (FI) was determined by the microplate reader (Victor Nivo multiplate reader, PerkinElmer) at excitation and emission wavelengths of 488 and 530 nm, respectively. The FI was divided by the OD₆₀₀ value to normalize the cytotoxicity with respect to the growth of the bacteria. Untreated cells were processed similarly and used as the control (42). The statistical significance was determined using Student’s *t*-test comparing the control and the treated sample.

#### Comparative microscopic analysis of cell viability by trypan blue staining

The antimicrobial effect of the iron chelator was investigated using the trypan blue staining method on *A. baumannii*, similar to the method described elsewhere. Briefly, cells in the mid-exponential phase (OD₆₀₀: 0.5) were harvested and washed with 1× PBS at 6,000 rpm in a centrifuge and the pellet was resuspended in 1× PBS to make the OD₆₀₀: 1.0. The cell suspension was then treated with several iron chelators (DIP, β-thujaplicin) and kept in a shaker incubator for 2 h at 37°C at 180 rpm. The treated cells were washed with 1× PBS and mixed with trypan blue for staining purposes. The stained cells were placed on a coverslip and observed under bright-field microscopy at 40× magnification. Trypan blue can penetrate the lysed cells, while live intact cells were stained along their periphery, as seen under the microscope.

#### Scanning electron microscopic study

Overnight-grown *A. baumannii* cells were freshly inoculated into LB at a 1:100 ratio and allowed to grow up to the mid-exponential phase (OD₆₀₀ = 0.5). Cells were harvested by centrifugation at 6,000 rpm for 10 min and washed with 10 mM PBS (pH 7.5). The bacterial culture was subsequently diluted in the same buffer to OD₆₀₀ = 1.0 (cell number 10⁹). Bacterial cells were treated with 200 µM β-thujaplicin and incubated for 2 h at 37°C with shaking at 180 rpm. The cells were harvested at 6,000 rpm for 10 min, and the pellet was washed with 10 mM PBS twice. Glutaraldehyde (2.5%) was used to fix the bacterial cells at 4°C overnight. Following centrifugation, the fixed cells were washed with 10 mM PBS again to remove the excess fixative from the sample. SEM analysis was performed on a Quanta FEG 250 operating at an accelerating voltage of 20 kV. Silicon wafers were used to prepare SEM specimens. For each sample, a droplet of bacterial suspension was cast on a silicon wafer and dried overnight in a vacuum desiccator. A gold coating was done before inserting the samples into the microscope (43).

#### Detection of persister cell generation

The overnight culture of *A. baumannii* was freshly inoculated into LB at a 1:100 ratio and grown up to the mid-exponential phase. Cells were diluted to 10⁸ CFU/mL, and added in fresh LB medium in 1:100 ratio and incubated at 37°C in an incubator shaker for 24 h. After incubation, planktonic cells were removed by washing twice with 1× PBS, followed by the addition of 5 mL of fresh LB to each tube with 10× MIC of β-thujaplicin and further incubated for another 24 h under the same conditions. After incubation, planktonic cells were first removed by washing three times with 1× PBS. To dislodge the adherent persister cells, the samples were sonicated in 5 mL of fresh 1× PBS. For 100 µL of the solution, 10× MIC of colistin was added as a control, while β-thujaplicin was added at its MIC, 4× MIC, and 8× MIC values, and samples were incubated for 6 and 12 h at 37°C. The number of persister cells was determined by plating on a nutrient agar plate (44). The result was plotted using GraphPad Prism software, and statistical analysis was performed with Student’s *t*-test.

#### Transcriptomic analysis

*A. baumannii* cells were grown up to the exponential phase and treated with sub-lethal dose (100 µM) of β-thujaplicin and incubated for 2 h in a shaker incubator at 37°C. After incubation, cells were harvested at 5,500 rpm for 10 min and washed with 1× PBS twice. Bacterial RNA isolation was performed using the GCC Biotech Kit (GR1001). The concentration of RNA was measured using a Nanodrop. A total of 1,000 ng of RNA was taken for cDNA synthesis, which was carried out using the GCC Biotech Kit (G4641A). Quantitative real-time PCR was done in a total of 10 µL reaction mixture. PCR amplification for cDNA synthesis was performed under the following conditions: 35 cycles with initial denaturation at 95°C for 5 min, followed by 95°C for 20 s, annealing at 55°C for 45 s, and a final extension at 72°C for 30 s. The mRNA expression level of each gene was normalized using the expression of the housekeeping gene (*rpsL*), and the relative fold change in gene expression was calculated using the 2^⁻ΔΔCT^ method. The analysis was performed in three biological replicates (45).

#### Rescue analysis

The rescue assay was conducted in accordance with previously outlined methodologies with minor modifications (46). The *A. baumannii* culture from overnight growth was introduced into LB medium at a 1:100 ratio. One tube served as a control, while another was treated with 100 µM β-thujaplicin. Following a growth period of 3 h (mid-exponential phase), the 100 µM β-thujaplicin-treated culture was divided into five tubes. While one tube remained as 100 µM β-thujaplicin, the remaining four tubes were subjected to a combination treatment at specific doses. Subsequently, four of the five tubes were supplemented individually with 25, 50, 75, and 100 µM FeSO₄. All tubes were maintained at 37°C with shaking at 180 rpm. Samples were taken from each set, and the optical density was measured at 600 nm using a Victor-Nivo multiplate reader for 9 h. After 2 and 4 h of successive FeSO₄ addition, bacterial culture samples were taken, and bacterial viability was determined by CFU. The recorded values were plotted from triplicate samples using GraphPad Prism.

#### MTT assay

The cytotoxic effect of β-thujaplicin was verified by assessing its effect on C2C12 (mouse myoblast cell line). Cells were first seeded into a 96-well culture plate and subjected to treatment with different concentrations (0.5, 1, 2, and 8× MIC) of β-thujaplicin for 24 h inside a CO₂ incubator at 37°C. After incubation, the medium was removed, and 5 µL of MTT solution was added from a 5 mg/mL stock. Then, cells were further incubated for 3 h, and 200 µL of DMSO was added to each well. The optical density of the cell suspension was measured at 570 nm using a multiplate reader. Cell viability expressed as a percentage of reduction was calculated using the following formula (47)


$$\%\;Cytotoxic\;=\frac{\left(UV\;absorbance\;of\;untreated\;cell\right)}{\left(UV\;absorbance\;of\;control\right)}\times 100.$$

## SUPPLEMENTAL MATERIAL

Supplemental figures aac.01689-24-s0001.pdf Figure S1 to S3.

ASM does not own the copyrights to Supplemental Material that may be linked to, or accessed through, an article. The authors have granted ASM a non-exclusive, world-wide license to publish the Supplemental Material files. Please contact the corresponding author directly for reuse.
