# Supplementary material for: Harnessing the effect of iron deprivation to attenuate the growth of opportunistic pathogen Acinetobacter baumannii
Source: Antimicrob Agents Chemother. 2025 Apr 9;69(5):e01689-24. doi: 10.1128/aac.01689-24 (PMC12057376; doi:10.1128/aac.01689-24)
Supplement: Supplemental figures — Figure S1 to S3. [file aac.01689-24-s0001.pdf]

Supplementary Figure 1

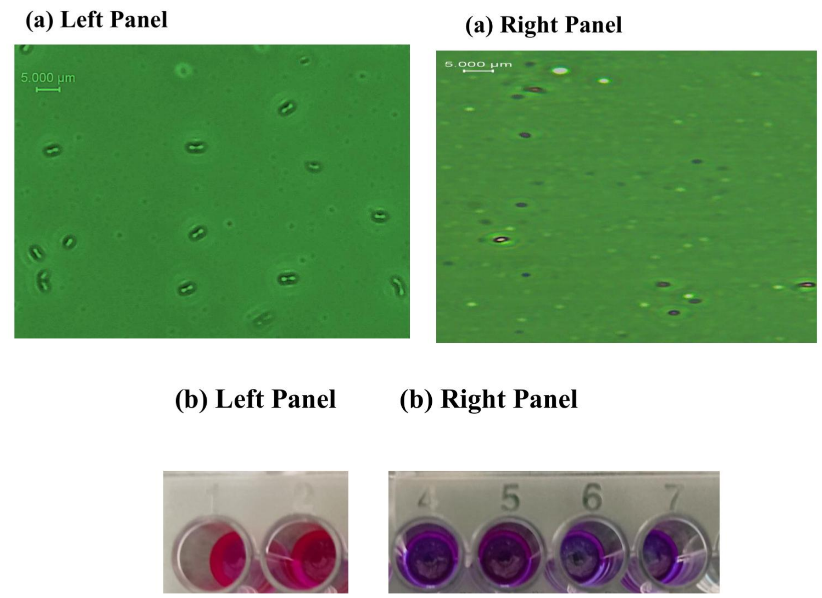

Supplementary Figure 1: Cell viability analysis: (a) The cell viability experiment was conducted using the Trypan blue staining method. In the control group (left panel), live cells are observed, as the cell membrane is stained with Trypan blue while the cytosol remains unstained due to an intact membrane. In contrast, cells treated with 100 µM β-Thujaplicin undergo lysis, resulting in pore formation on the outer membrane, allowing the dye to easily penetrate the cells (right panel). **(b)** Cell viability assessed using resazurin staining showed that in the control group, where no treatment was applied, the metabolic enzymes of live cells converted resazurin to pink. However, in cells treated with 100 and 200 µM β-Thujaplicin, the resazurin color remained unchanged, indicating cell death. All images were captured within a 5 µM range.

Supplementary Figure 2

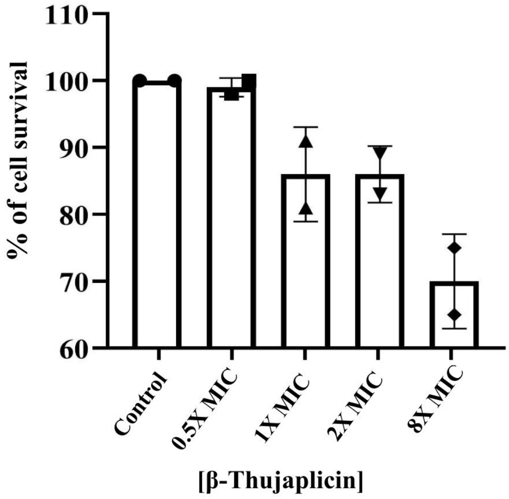

Supplementary Figure 2: Toxicity analysis: The mammalian cell cytotoxicity assay was performed to evaluatethe cytotoxic effect of β-Thujaplicin using a concentration eight times the MIC, which resulted in 25% cell death compared to control.

Supplementary Figure 3

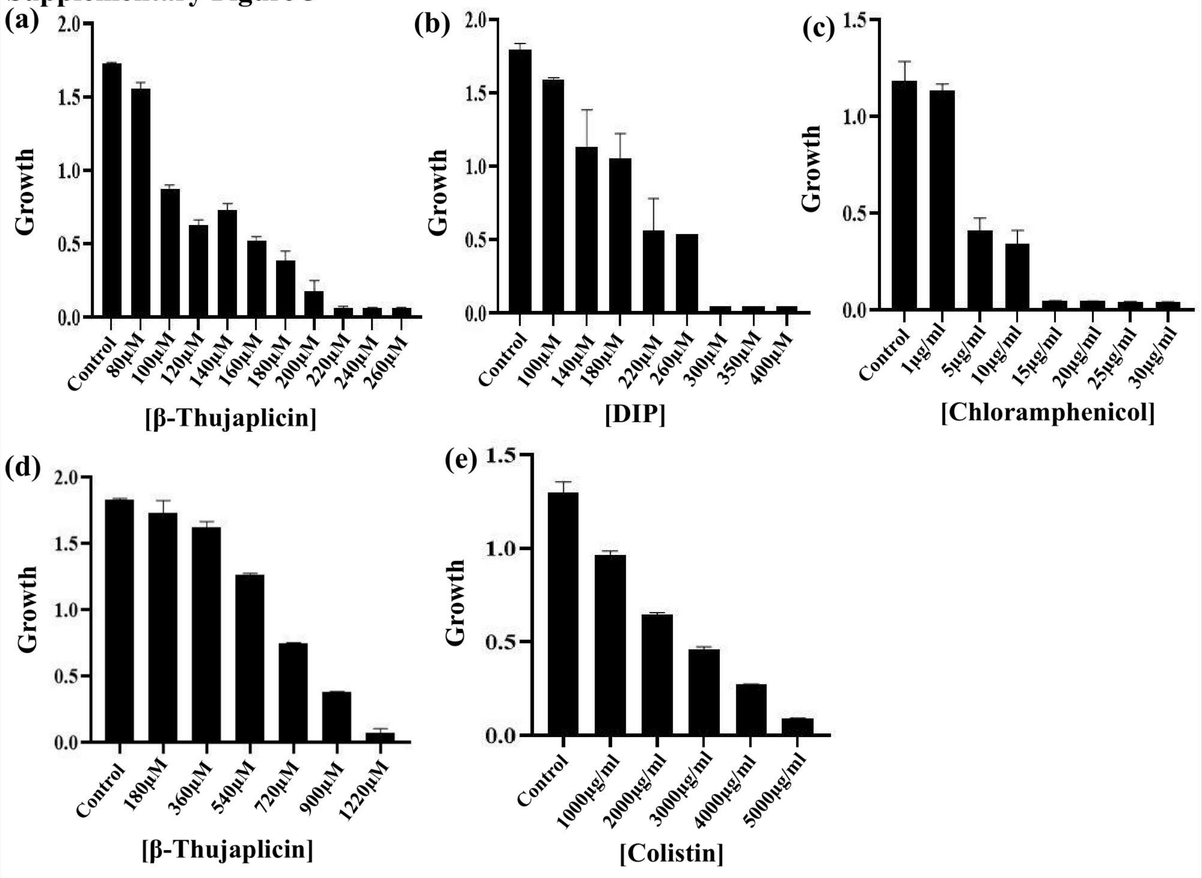

Supplementary Figure 3: Effect of iron chelator and antibiotics on wild type *Acinetobacter baumannii*, and clinical strain *Pseudomonas aeruginosa:* **(a)** The MIC of β-Thujaplicin for wild type strain of *A. baumannii* (DS002) is approximately 200 µM. **(b)** MIC value of DIP for *A. baumannii* is 300 µM. **(c)** The MIC range of chloramphenicol for *A. baumannii* is 15 µg/ml. **(d)** Clinical strain of *Pseudomonas aeruginosa* (PA1114) shows higher MIC value i.e. 1220 µM. **(e)** The MIC of colistin for Multidrug resistant *P.aeruginosa* is 5000 µg/ml.
